# When lightning strikes the heart: a rare injury with features of non‑ST elevation myocardial infarction – a case report

**DOI:** 10.1186/s43044-025-00632-4

**Published:** 2025-03-31

**Authors:** Muhammad Isra Rafidin Rayyan, Aulia Shakila Rahma, Nabil Athoillah, Muhammad Daffa Husniatama, Nurwahyudi Nurwahyudi

**Affiliations:** 1 Faculty of Medicine, University of Jember, Jember, Indonesia; 2 Cardiovascular Department, dr. H. Koesnadi General Hospital, Bondowoso, Indonesia

**Keywords:** Cardiac injury, Case report, Electrocardiography, Lightning strike, Non-ST elevation myocardial infarction

## Abstract

**Background:**

Injuries from lightning strikes are infrequent but potentially life-threatening, mainly caused by indirect electrical injuries. Although various organ systems can be involved, the effects of electric current on the cardiovascular system can manifest as electrocardiographic (ECG) changes, elevated cardiac-specific enzymes, and cardiac and respiratory arrest, although rare. This case report aims to describe the ECG changes that occur due to lightning strikes and resemble the features of non-ST elevation myocardial infarction (NSTEMI).

**Case presentation:**

A 31-year-old man with no history of cardiovascular disease risk factors other than being an active smoker presented to the emergency department after being unconscious for 15 min due to a lightning strike while digging a grave. He complained of headache, tingling in the extremities, and general weakness. The physical examination found no burns or bruises. The initial ECG examination showed inferior ST depression with elevated troponin-I, which led to the diagnostic features of NSTEMI. When a repeat ECG examination was performed the next day, an inverted T wave was found in the inferior leads. Echocardiographic examination revealed only concentric left ventricular hypertrophy and mild diastolic dysfunction without kinetic abnormalities. Because it was assumed that the NSTEMI features were not due to an atherothrombotic process, the patient was treated conservatively without worsening his condition.

**Conclusions:**

Lightning strikes can cause a range of cardiac injuries, from mild ECG changes to fatal damage. Early recognition of lightning injury syndrome and close monitoring of complications through signs and symptoms, ECG, cardiac enzymes, and echocardiography are crucial for improving patient outcomes.

## Background

Lightning strike injuries are one of the unique subtypes of electrical injuries. Every year, there are approximately 1.4 billion lightning strikes worldwide. Of these, approximately 24.000 strikes cause significant morbidity and mortality. The worldwide annual mortality rate from lightning strikes ranges from 0.2 to 1.7 cases per one million population [1, 2]. In general, injuries from lightning strikes can occur in various ways, including (1) direct strikes, which are most often fatal; (2) contact injuries between the victim and conducting elements such as metals; (3) ground currents, when lightning travels from the point of strike through the ground and into the victim's body; and (4) injuries due to explosive effects [3].

Although injuries from lightning strikes are rare, lightning can carry over 1 million volts with very large currents ranging from 30.000 to 200.000 amperes and is classified as a high-voltage injury [4]. This high voltage and rapid release of energy can cause a wide variety of injuries, including burns, neurological deficits, and multisystem organ failure. One of the most severe complications of lightning strike injuries is cardiovascular damage that can manifest as arrhythmias, myocardial injury, pericardial complications, aortic dissection, and cardiac arrest [5, 6].

We report a case of a patient presenting with a unique presentation of electrocardiographic (ECG) changes resembling non-ST-segment elevation myocardial infarction (NSTEMI) following a lightning strike. This case report aims to discuss the diagnostic and therapeutic challenges associated with the case and provide insight into the complex pathophysiology of electrical injury to the heart while emphasizing the importance of close monitoring in patients exposed to lightning strikes. This case report is based on the CARE (for CAse REports) 2013 guidelines [7].

## Case presentation

A 31-year-old male patient presented to the emergency department after being unconscious for 15 min due to a lightning strike. The patient claimed he was digging a grave with four friends when the incident occurred. When he arrived at the hospital, he was fully conscious but complained of headache, tingling in all four extremities, and general weakness. He denied having any risk factors for cardiovascular disease, such as hypertension, diabetes mellitus, dyslipidemia, heart disease, and previous similar complaints, but he was an active smoker.

The physical examination revealed Glasgow Coma scale (GCS) E4V5M6, blood pressure 110/80 mmHg, heart rate 91 beats/min, respiratory rate 20 breaths/min, body temperature 36.2 °C, and SpO2 98% with oxygen 3 L/min. There were no abnormalities in the examination of the respiratory and cardiovascular systems. The abdominal examination was also within normal limits; no edema was found in the limb examination, and there were no burns or bruises on the skin.

**Fig. 1:**
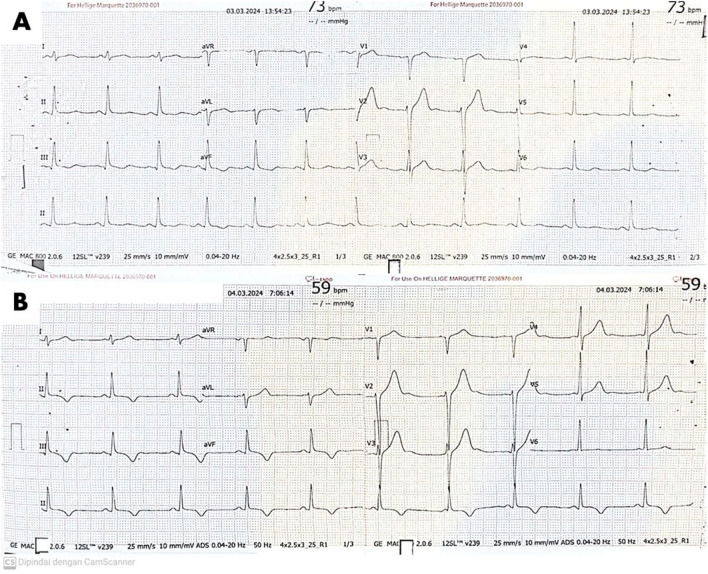
ECG examination results **A** at the time of arrival and **B** one day later showing T-inversions

The initial 12-lead ECG examination in the emergency room showed sinus rhythm, 73 beats/minute, normal axis, and downsloping ST depression in the inferior leads (II, III, aVF). A repeat ECG examination on the following day showed sinus rhythm, 59 beats/minute, normal axis, and T-inversion in the inferior leads (Fig. 1). The chest radiographs and laboratory examination revealed no significant findings, except for mild hypokalemia of 3.1 mmol/L (normal value: 3.6–5.5) and positive troponin-I (normal value: negative). Transthoracic echocardiographic examination revealed a left ventricular ejection fraction (LVEF) of 57.22%, concentric left ventricular hypertrophy (LVH), mild diastolic dysfunction, and no regional wall motion abnormalities.

Based on the examination results, he was diagnosed with cardiac injury due to lightning strikes and mild hypokalemia with a differential diagnosis of NSTEMI. He was then consulted by a cardiologist and hospitalized in the intensive coronary care unit (ICCU). As the NSTEMI was atypical and most likely not caused by an atherothrombotic process, and there was no access to coronary angiography at our hospital, he was not given standard treatment for acute coronary syndromes, such as antiplatelets and anticoagulants. He was treated conservatively with ringer lactate infusion 500 ml/24 h, ramipril 2.5 mg once daily, bisoprolol 2.5 mg once daily, trimetazidine 35 mg twice daily, and coenzyme Q10 60 mg once daily. His clinical course during hospitalization was satisfactory, as there were no symptoms and his vital signs were stable; he was transferred to the regular inpatient room on the third day and discharged the next day.

## Discussions

Lightning strikes are a natural phenomenon with unique properties that set them apart from other electrical injuries. Lightning injuries are classified as high-voltage injuries because they can expose the body to more than 1 million volts. These injuries are most common in young men engaged in outdoor activities [8]. Factors determining the severity of electrical injury include voltage, resistance to electricity, type of current (direct or alternating), duration of exposure, path of current through the body, and amount of energy delivered [9]. The most essential difference between lightning and other electrical injuries is the duration of exposure. Victims can usually be exposed to lightning for a short period, between 1/1000 and 1/10 of a second. Due to this short exposure period, little energy is transferred internally. In contrast, most energy flows externally to the victim's body, also known as the “flashover” effect, so a person can generally survive a lightning strike [4, 10].

The human body has an internal resistance to electricity of around 500 Ohms, but there are significant variations in different tissues. Skin, bone, and fat have a higher resistance to electricity, while nerves and blood vessels have a lower resistance and thus conduct electricity more easily [9]. Based on the mechanism, lightning strike injuries can be classified as direct strikes, side splashes, contact injuries, and ground strikes. Most injuries occur outdoors, whereas ground strike or side splash injuries are the most common. About 50% of injury cases occur due to ground strikes, while side splash injuries contribute about 30%. In ground strike injuries, lightning strikes the ground and passes it to the victim. In side splash injuries, lightning strikes a nearby object, such as a tree, and then strikes the victim [4].

Meanwhile, direct strikes only account for 3 to 5% of cases, and contact injuries only 1–2%. Although direct strike injuries are rare, they have the highest fatality rate because the lightning strikes the victim directly. In contrast, in contact injuries, the lightning strikes another object, such as metal touched by the victim. Although rare, patients can also be injured by extreme temperatures or blast waves generated by lightning where strike temperatures have been reported to reach 30.000 °C [1, 2, 4, 10].

The heart is one of the organs most often affected by electrical injury because electric current generally follows the blood flow axis due to its low resistance, including the nervous system. The two main complications to the cardiovascular system from lightning strikes are arrhythmias and myocardial injury [9]. In general, the cardiovascular manifestations of lightning strikes involve electrical current disturbances, depolarization of the myocardium, catecholamine-induced stress responses, tissue damage, and vascular injury [2, 6].

The mechanisms behind electrical-induced cardiac arrhythmias are not fully understood. However, biopsies report arrhythmogenic foci in patchy myocardial fibrosis with increased numbers of Na + and K + pumps and may trigger re-entry arrhythmias. Cardiac injury and myocardial ischemia can usually result from coronary artery vasospasm and extensive release of catecholamines, and secondarily from arrhythmias that cause a decrease in coronary artery blood flow. Sudden death after a lightning strike is generally caused by simultaneous cardiac and respiratory arrest and is more common with direct strikes. Typically, patients are more likely to experience asystolic cardiac arrest than ventricular fibrillation due to the simultaneous depolarization of all myocardial cells [8–10]. Meanwhile, vascular injury may result from compartment syndrome or electrical coagulation of small blood vessels, although this is rare after a lightning strike. Damage to the arterial endothelium can be instantaneous or delayed for several weeks, leading to thrombosis and tissue necrosis [6, 8].

Due to the high incidence of dysrhythmias and autonomic dysfunction following lightning strikes, continuous cardiac monitoring is recommended unless the patient meets all of the following criteria: (1) low-voltage exposure; (2) asymptomatic; (3) no history of loss of consciousness; (4) normal physical examination (no burns or contact injuries) [8]. Cardiac monitoring for 12 to 24 h after presentation is recommended for patients suspected of high-voltage exposure, even if they do not have any obvious symptoms. Serial ECG examinations are mandatory for heart rhythm disturbances such as QT prolongation, T wave inversion, non-specific ST segment, and T wave changes [4]. Echocardiography, especially for patients who experience chest pain, whether accompanied by dyspnea or not, is performed to evaluate changes in the structure and function of the heart. ECG changes after a lightning strike may be present without a significant decrease in motility and may also not occur in cases of structural damage [2, 8]. Cardiac enzymes can also be used as a routine screening to support the previous two examinations. Some experts claim troponin levels and echocardiography can detect myocardial injury after electrical exposure. However, others have found that the significance of troponin for assessing prognosis remains controversial, although serial troponin examinations are usually performed in practice [8].

On the other hand, coronary angiography is not routinely performed because pathogenetically, lightning-induced cardiac injury is believed to occur due to coronary vasospasm rather than atherothrombotic mechanisms [10, 11]. In addition, there are no data to support the use of coronary angiography for diagnostic purposes in this case. Several previous case reports have also revealed that there is no evidence of coronary artery occlusion, even in patients who have died from cardiac arrest after being struck by lightning [3, 6, 11, 12].

In our patient, after being struck by lightning, he claimed to have lost consciousness for 15 min. Then, on examination, ECG changes in T-inversion in the inferior leads accompanied by an increase in troponin-I were obtained, supporting the diagnostic criteria of NSTEMI. During hospitalization, the patient denied complaining of cardiac chest pain. Interpreting these findings can be challenging as there is not always a correlation between ECG changes, elevated cardiac enzymes, and the patient's clinical symptoms. In addition, since no features suggestive of ischemic heart disease were found on echocardiographic examination, it can be believed that the cardiovascular manifestations in the patient were more likely due to non-obstructive coronary artery disease.

The management of lightning injuries should be individualized according to the patient's presentation and organ system involvement. Treating lightning strike victims should follow standardized guidelines according to Advanced Trauma Life Support (ATLS) [4, 6]. Most lightning strike victims who appear healthy without vital sign abnormalities or injuries requiring hospitalization can be safely discharged if they do not have a high-risk history, such as suspected direct strike, loss of consciousness, focal neurological deficits, chest pain, shortness of breath, or significant burns. Patients affected by lightning strikes may appear to be dead because they have paralysis of central respiratory control, dilated and unreactive pupils, and cardiac arrest [3]. However, in these cases, aggressive care should be focused on those who appear dead, called a “reverse triage” system, as prolonged resuscitation efforts may have a higher success rate in lightning strike victims than cardiac arrest from other causes [10, 12].

**Fig. 2:**
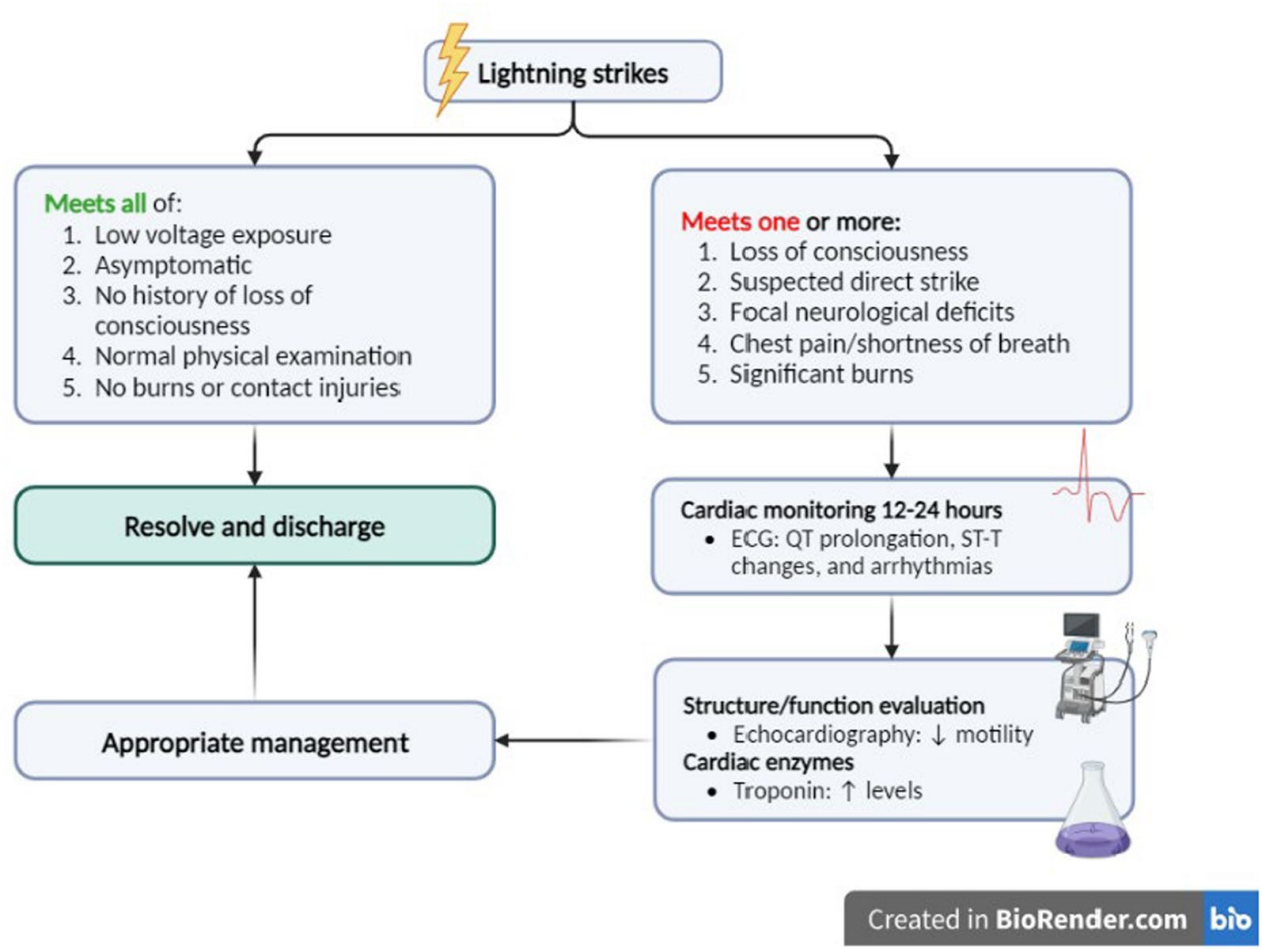
Summary of management algorithm for lightning strike patients (created in BioRender.com)

To date, the optimal management of myocardial injury due to electric shock, particularly lightning strikes, remains a challenge as there has yet to be a clear consensus regarding treatment options in such patients. Most importantly, cardiovascular manifestations after lightning strikes generally do not require therapy, such as acute coronary syndrome, because the myocardial damage that occurs is believed to be caused by coronary vasospasm rather than coronary atherothrombosis. Some literature states that the ECG changes that occur will disappear spontaneously within a few days so that patients do not require antiplatelet and anticoagulant administration despite evidence of elevated cardiac enzymes [4, 6, 13]. A summary of the lightning strike patient management algorithm can be seen in Fig. 2.

In this case, since the patient was young and had no significant cardiovascular risk factors, he was only given supportive medications such as angiotensin-converting enzyme (ACE) inhibitors, beta-blockers, trimetazidine, and coenzyme Q10. This aligns with Celebi et al., who also reported good results from administering ACE inhibitors and beta-blockers as anti-remodeling in patients with inferior ST-segment elevation myocardial infarction (STEMI) after electric shock [14]. Furthermore, trimetazidine is an anti-ischemia agent that prevents the β-oxidation of fatty acids, increases glucose oxidation, and reduces the intracellular accumulation of hydrogen ions, lactate, sodium, and calcium ions [15]. Meanwhile, coenzyme Q10 is a quinone-derived compound produced naturally in mitochondria. Coenzyme Q10 supplementation provides pleiotropic effects in heart disease through its role as an anti-inflammatory, antioxidant, membrane stabilizer, and anti-remodeling agent [16]. Therefore, in this case, both drugs were used as adjunctive therapy due to their cardioprotective effects.

## Conclusions

Lightning strike injuries can damage most organs of the body, which can lead to life-threatening conditions. The heart is one of the most commonly affected organs as it has a low resistance to electric current and can manifest as mild ECG changes, malignant arrhythmias, myocardial injury, and cardiac arrest. This case report highlights the unique findings of ECG changes following lightning strikes that resemble the diagnostic features of NSTEMI. Early recognition and close observation through ECG, cardiac enzymes, and echocardiography are recommended in high-risk patients to guide treatment and improve patient outcomes.

## Data Availability

No datasets were generated or analyzed during the current study.

## Abbreviations

ACE: Angiotensin-converting enzyme
ATLS: Advanced trauma life support
ECG: Electrocardiographic
GCS: Glasgow Coma scale
ICCU: Intensive coronary care unit
LVEF: Left ventricular ejection fraction
LVH: Left ventricular hypertrophy
NSTEMI: Non-ST elevation myocardial infarction
STEMI: ST-segment elevation myocardial infarction
